# Association of a Deletion of *GSTT2B* with an Altered Risk of Oesophageal Squamous Cell Carcinoma in a South African Population: A Case-Control Study

**DOI:** 10.1371/journal.pone.0029366

**Published:** 2011-12-27

**Authors:** Marco Matejcic, DongPing Li, Natalie J. Prescott, Cathryn M. Lewis, Christopher G. Mathew, M. Iqbal Parker

**Affiliations:** 1 International Centre for Genetic Engineering and Biotechnology, Cape Town Component, Observatory, Cape Town, South Africa; 2 Division of Medical Biochemistry and IIDMM, UCT Faculty of Health Sciences, Cape Town, South Africa; 3 Department of Medical and Molecular Genetics, King's College London, King's Health Partners, Guy's Hospital, London, United Kingdom; 4 Social, Genetic and Developmental Psychiatry Centre, Institute of Psychiatry, King's College London, London, United Kingdom; Ohio State University Medical Center, United States of America

## Abstract

**Background:**

Polymorphisms in the Glutathione S-transferase genes are associated with altered risks in many cancers, but their role in oesophageal cancer is unclear. Recently a 37-kb deletion polymorphism of *GSTT2B* that reduces expression of *GSTT2* has been described. We evaluated the influence of the *GSTT1* and *GSTT2B* deletion polymorphisms, and the *GSTP1* Ile105Val polymorphism (rs1695) on susceptibility to oesophageal squamous cell carcinoma (OSCC) in the Black and Mixed Ancestry populations of South Africa.

**Methods and Results:**

The *GSTT1*, *GSTT2B* and *GSTP1* variants were genotyped in 562 OSCC cases and 907 controls, and tested for association with OSCC and for interaction with smoking and alcohol consumption. Linkage disequilibrium (LD) between the deletions at *GSTT1* and *GSTT2B* was determined, and the haplotypes tested for association with OSCC. Neither the *GSTT1* deletion nor the *GSTP1* Ile105Val polymorphism was associated with OSCC risk in the Black or Mixed Ancestry populations. The *GSTT2B* deletion was not associated with OSCC risk in the Black population, but was associated with reduced risk of OSCC in the Mixed Ancestry population (OR = 0.71; 95% CI 0.57–0.90, p = 0.004). Case-only analysis showed no interaction between the *GS*T polymorphisms and smoking or alcohol consumption. LD between the neighboring *GSTT1* and *GSTT2B* deletions was low in both populations (r^2^_Black_ = 0.04; r^2^_MxA_ = 0.07), thus these deletions should be assessed independently for effects on disease risk.

**Conclusions:**

Although there was no association between the *GSTT1* deletion polymorphism or the *GSTP1* Ile105Val polymorphism with OSCC, our results suggest that the presence of the recently described *GSTT2B* deletion may have a protective effect on the risk of OSCC in the Mixed Ancestry South African population. This is the first report of the contribution of the *GSTT2B* deletion to cancer risk.

## Introduction

Oesophageal cancer is the third most common malignancy of the digestive tract and the seventh leading cause of cancer-related deaths worldwide [1]. Cancer of the esophagus exists in two main forms with different etiological and pathological characteristics: oesophageal squamous cell carcinoma (OSCC) and oesophageal adenocarcinoma (OADC). OSCC is the predominant histological subtype in developing countries [2] and occurs at higher frequencies in Southern and Eastern Africa, Northern China and the central Asian belt from Turkey through Iran, Iraq and Kazakhstan [3]–[6]. In South Africa it is one of the most common malignancies and the most common cancer among Black males, with a variable incidence rate among regions [7]. The regional difference in the frequency of OSCC is probably due to genetic polymorphism and variable exposure to environmental factors, including nutritional factors that are associated with the development of this disease [8]. Epidemiological studies have also identified smoking and alcohol consumption as major risk factors associated with OSCC [9]–[11]. Our studies have shown that genetic polymorphisms in the alcohol metabolizing enzymes (ADH2*1 and ADH3*2) and detoxification enzymes (*SULT1A1*, *CYP3A5* and *P450 2E1*) are associated with susceptibility to OSCC in the South African population [12]–[15].

**Figure 1:**
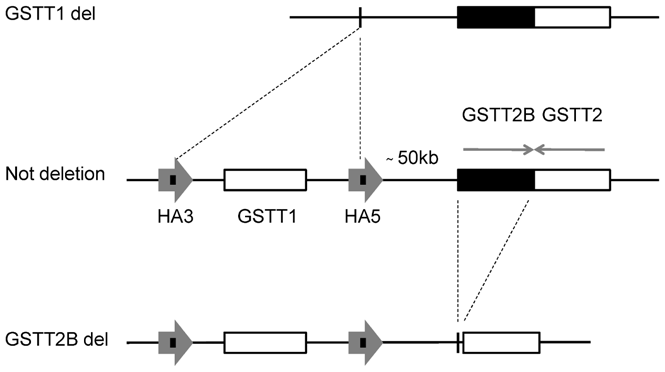
Deletion polymorphisms of the *GSTT1* and *GSTT2B* genes. The *GSTT1* and *GSTT2* genes are part of the Theta-class GST gene cluster on chromosome 22q11.23. *GSTT1* and *GSTT2* are separated by approximately 50 kb and share 55% sequence homology. *GSTT1* is flanked by two 18 kb regions, HA5 and HA3, which are more than 90% homologous. In their central portion, HA3 and HA5 share a 403-bp sequence with 100% identity. HA5 and HA3 are direct repeats. The *GSTT1* null allele arises by homologous recombination of the left and right 403-bp repeats, which results in a 54-kb deletion containing the entire *GSTT1* gene. *GSTT2* is positioned adjacent to the HA5 repeat. The duplicate copy of *GSTT2* is a pseudogene named *GSTT2B*, because of an abnormal exon 2/intron 2 splice site that causes a premature translation stop. *GSTT2* and *GSTT2B* are inverted repeats. The deletion of *GSTT2B* has a strong influence on *GSTT2* gene expression, as described in the text.

The Glutathione S-transferases (GSTs) are the major phase II metabolizing enzymes and play a key role in cellular detoxification of toxic compounds such as tobacco-related carcinogens [16]. Specifically, GSTs catalyze the conjugation of glutathione (GSH) to a wide variety of substrates with electrophilic functional groups including the products of oxidative stress, environmental pollutants and carcinogens, thereby neutralizing their electrophilic sites and producing stable and soluble compounds easily excreted from the organism [17]. Homozygous deletion of GST genes that results in a total lack of the enzyme activity is a common polymorphism in humans [18]. The *GSTT1* and *GSTT2* genes are located at a recombination-prone locus on chromosome 22q11.23 separated by about 50 kb [19], [20]. *GSTT1* is flanked by two highly homologous regions (HA3 and HA5) that share an identical 403-bp repeat (Fig. 1). A homologous recombination event involving the HA5 and HA3 403-bp repeats results in a 54-kb deletion containing the entire *GSTT1* gene resulting in a lack of *GSTT1* activity [21]. The *GSTT2* gene is part of a 64-kb inverted repeat (DNA-IR), the other copy of which is located immediately adjacent to *GSTT2* and contains the homologous pseudogene, *GSTT2B*, which shares 98% sequence identity with *GSTT2* (Fig. 1). A recent study [22] reported that a 37-kb deletion of the proximal repeat encompassing the *GSTT2B* gene reduces expression of the *GSTT2* gene by more than 80% in cell lines homozygous for the deletion. The frequency of *GSTT1* and *GSTT2B* deletions varies between populations. Homozygous deletions of *GSTT1* are present in 15–20% of Whites, 45–64% of Asians and 20–41% of Sub-Saharan Africans [23]–[27]. The *GSTT2B* homozygous deletion occurs in approximately 42% of Whites, 29% of Japanese/Chinese and 18% of the Yoruban population of West Africa [22]. The *GSTP1* gene is located on chromosome 11q13 and is the major GST expressed in the oesophagus [28]. *GSTP1* contains a single nucleotide polymorphism (SNP) at position 313 (A>G) that substitutes Isoleucine (Ile) for Valine (Val) at amino acid position 105, resulting in an enzyme with altered substrate affinity and decreased enzyme activity [29]. Approximately 29% of Whites and 15% of Asians are carriers of the *GSTP1* Val105 variant allele [30]–[33], and the frequency varies from 16% to 24% among Southern and Eastern Africans. In South African Xhosa the frequency was reported as 53%, which is significantly higher than that reported for other African ethnic groups [27].

The importance of glutathione conjugation by GSTs enzymes in carcinogen detoxification and drug metabolism led to extensive studies of polymorphisms in these genes as potential risk factors for different types of cancer [18], [34]. In oesophageal cancer, meta-analysis showed that the *GSTT1* deletion allele was not associated with increased risk of oesophageal cancer in Whites and Asians, but was associated with a reduced risk of OSCC in the Brazilian population [35], [36]. A recent meta-analysis reported association of the *GSTP1* Val105 allele with increased risk of OSCC in Whites [37], and we previously reported association of the *GSTP1* Ala114Val but not Ile105Val SNP with an increased risk of OSCC in the South African population [15].

In view of the somewhat conflicting nature of previous reports, and the new evidence of an important functional deletion at the *GSTT1/2* locus, we have investigated the relationship between the *GSTT1* and *GSTT2B* deletions and the *GSTP1* Ile105Val polymorphism and susceptibility to OSCC in the South African Black and Mixed Ancestry populations. Features of the study design include a much larger sample size than in previous work to provide greater statistical power, separate analysis of the Black and Mixed Ancestry data to avoid confounding the analysis by differences in genetic structure, and the use of multiplex PCR assays which differentiate heterozygotes from homozygotes for the *GSTT1* and *GSTT2B* deletions, thus allowing full resolution of all 3 possible genotypes.

Using the above genotyping assays, we successfully genotyped the *GSTT1*, *GSTT2B* and *GSTP1* variants in 562 OSCC cases and 907 controls, and tested for association with OSCC.

The risk of OSCC has been associated with smoking and alcohol consumption in multiple studies, and we therefore tested for a gene-environment interaction in a case-only analysis, stratifying cases by smoking and alcohol consumption status.

This is, to our knowledge, the largest investigation to date of the association of GST functional polymorphisms with cancer in African populations, and the first to assess the role of the *GSTT2B* deletion in cancer risk.

## Results

### *GSTT1* deletion analysis

**Figure 2:**
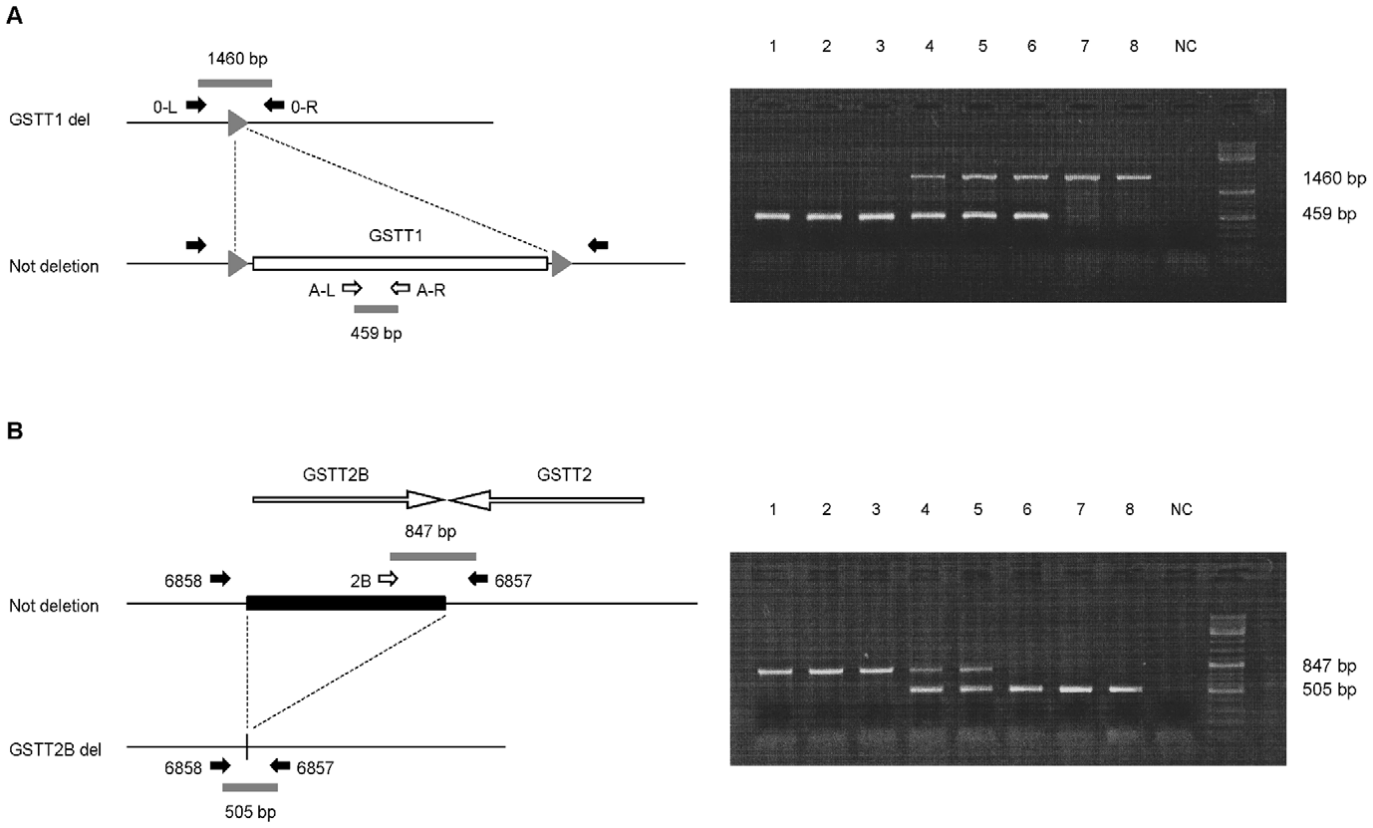
PCR assay for *GSTT1* and *GSTT2B* deletion genotyping. A four primer set was used in a single reaction to determine the presence or absence of the *GSTT1* allele. (A); Primers A-L and A-R amplify a 459 bp sequence present in the *GSTT1* gene, while primers O-L and O-R amplify a joint sequence of 1460 bp resulting from the deletion of *GSTT1*. When *GSTT1* is not deleted, the DNA fragment (*GSTT1*) between the forward (O-L) and reverse primers (O-R) is too long for amplification and only the 459 bp sequence is amplified. Conversely, when *GSTT1* is deleted, the 459 bp sequence is not amplified because absent while the 1460 bp fragment is now amplified. As a result of the PCR reaction, the only presence of a 459 bp band (lines 1–3 in the gel picture) or of a 1460 bp band (lines 7, 8) defines the *WT/WT* and the *del/del* genotypes respectively; while the presence of both fragments defines the heterozygous genotype (lines 4–6). Lane 9 was used as negative control. Solid lines represent genomic sequences; white rectangle represents the deleted sequence; small gray triangles indicate the 408 bp repeats flanking the *GSTT1* gene. Expected PCR products are drawn as small gray bars. (**B**); A three primer set is used to determine the deletion polymorphism of *GSTT2B* in a single reaction. Primers 2B and 6857 amplify a 847 bp fragment detecting the presence of the *GSTT2B* gene, while primers 6857 and 6858 amplify a 505 bp sequence resulting from the deletion of *GSTT2B*. The 6857 primer is used for amplification of both fragments. When *GSTT2B* is not deleted, the DNA fragment (*GSTT2B*) between the 6857 and 6858 primers is too long for amplification and only the 847 bp fragment is amplified. Conversely, when the gene is deleted, the 505 bp fragment is amplified and the 847 bp fragment is not amplified. As a result of the PCR reaction we can have three possible polymorphisms: one single 847 bp (lines 1–3 in the gel picture) or one single 505 bp band (lines 6–8) represent the *WT/WT* and the *del/del* polymorphisms rispectively; while the contemporary presence of both bands represents the *WT/del* polymorphism (lines 4, 5). Lane 9 was used as a negative control. Solid lines represent genomic sequences; black rectangle represents the deleted sequence. Expected PCR products are drawn as small gray bars.

The genotype and allele frequencies for the *GSTT1* deletion polymorphism in the two South African populations were determined by multiplex PCR assay [21], which allows resolution of all 3 possible genotypes (Figure 2A). The results are shown in Table 1. No significant deviation from Hardy-Weinberg Equilibrium was found in Black controls; a marginal deviation was observed among Mixed Ancestry controls, which is not significant after correcting for multiple testing across the population groups and polymorphisms (p_Black_ = 0.359; p_MxA_ = 0.038). In order to investigate whether this deviation was due to genotyping error, 20% of samples were re-genotyped. There was no discrepancy between the two genotyping results, suggesting that the deviation from HWE was not due to genotyping error. The Mixed Ancestry population in South Africa is a heterogeneous ethnic group originating from the union of Europeans and Asians with several African populations, including the Xhosa [38]. Differences in the frequency of the deletion allele in these various ethnic groups [23]–[27] in combination with non-random mating may explain the modest deviation from HWE observed in this study. The *GSTT1* deletion was not associated with OSCC in either population (Table 1). The frequency of the *GSTT1* deletion allele in Black controls (55%) was similar to that reported for the Yoruban population (57%), with 30% of individuals being homozygous for the deletion. In the Mixed Ancestry controls the allele frequency (49%) was lower than that reported for Asian populations (65%) and higher than in Whites (33%), which is consistent with the origins of this population, with 22% of individuals being homozygous for the deletion [22]. A case-only analysis of smoking and drinking did not detect any interaction between environmental risk factors and the *GSTT1* deletion polymorphism in either the Black or Mixed Ancestry populations (Table 2). In the Mixed Ancestry population, the small number of smokers (N = 13) was below the threshold for a meaningful case-only analysis (see Methods).

**Table 1:** Genotype and allele frequencies of *GSTT1*, *GSTT2B* and *GSTP1* polymorphisms and association with OSCC in Black and Mixed Ancestry populations.

Gene	Study group		Genotype frequency			Allele frequency	Oesophageal squamous cell carcinoma	
	Population	Samples					OR (95% CI)	*p*-value
***GSTT1***			**Wt/Wt**	**Wt/Del**	**Del/Del**	**Del**		
	Black Controls	462	87 (19%)	238 (51%)	137 (30%)	0.554		
	Black Cases	311	59 (19%)	147 (47%)	105 (34%)	0.574	1.08 (0.88–1.33)	0.441
	MxA Controls	414	95 (23%)	228 (55%)	91 (22%)	0.495		
	MxA Cases	217	51 (23%)	118 (54%)	48 (22%)	0.493	0.99 (0.79–1.25)	1
***GSTT2B***			**Wt/Wt**	**Wt/Del**	**Del/Del**	**Del**		
	Black Controls	461	187 (40%)	206 (45%)	68 (15%)	0.371		
	Black Cases	320	140 (44%)	145 (45%)	35 (11%)	0.336	0.86 (0.69–1.06)	0.156
	MxA Controls	425	106 (25%)	212 (50%)	107 (25%)	0.501		
	MxA Cases	226	83 (37%)	97 (43%)	46 (20%)	0.418	0.71 (0.57–0.90)	0.004
***GSTP1***			**Ile/Ile**	**Ile/Val**	**Val/Val**	**Val**		
	Black Controls	474	100 (21%)	242 (51%)	132 (28%)	0.534		
	Black Cases	325	79 (24%)	155 (48%)	91 (28%)	0.518	0.94 (0.77–1.15)	0.547
	MxA Controls	428	145 (34%)	191 (45%)	92 (21%)	0.438		
	MxA Cases	229	69 (30%)	112 (49%)	48 (21%)	0.454	1.07 (0.85–1.34)	0.576

**OR** = odds ratio for the allele listed in the Allele Frequency column.

**Wildtype (Wt)** = non-deletion allele; **Del** = deletion allele.

**Table 2:** Association of *GSTT1*, *GSTT2B* and *GSTP1* polymorphisms with OSCC by smoking and drinking status in Black and Mixed Ancestry cases.

Gene	Population	Number of Patients		Test Allele	Oesophageal squamous cell carcinoma	
		Smokers	Non smokers		OR (95% CI)	*p*-value
*GSTT1*	Black	198	113	Del	1.18 (0.84–1.63)	0.335
	Mixed Ancestry	204	13	Del	-	-
*GSTT2B*	Black	205	115	Del	1.17 (0.83–1.66)	0.358
	Mixed Ancestry	215	11	Del	-	-
*GSTP1*	Black	210	115	Val	0.98 (0.71–1.35)	1
	Mixed Ancestry	216	13	Val	-	-

Gene	Population	Number of Patients		Test Allele	Oesophageal squamous cell carcinoma	
		Drinkers	Non drinkers		OR (95% CI)	*p*-value
*GSTT1*	Black	192	119	Del	1.17 (0.84–1.62)	0.350
	Mixed Ancestry	177	40	Del	1.24 (0.76–2.01)	0.393
*GSTT2B*	Black	200	120	Del	1.30 (0.92–1.83)	0.136
	Mixed Ancestry	186	40	Del	0.80 (0.49–1.30)	0.375
*GSTP1*	Black	204	121	Val	1.01 (0.74–1.39)	1
	Mixed Ancestry	189	40	Val	1.08 (0.67–1.76)	0.742

**OR** = odds ratio for the test allele.

**Smokers** = current and former smokers; **Drinkers** = light to heavy drinkers.

### *GSTT2B* deletion analysis

The PCR assay for the *GSTT2B* deletion is illustrated in figure 2B, and the genotype and allele frequencies are shown in Table 1. The observed genotype counts are comparable with expected and no deviation from HWE was observed in either control group (p_Blacks_ = 0.362; p_MxA_ = 1). The The *GSTT2B* deletion was not associated with OSCC in the Black population, although the frequency of the deletion allele was lower in cases than in controls. In the Mixed Ancestry population the *GSTT2B* deletion allele was significantly less frequent in cases than in controls, and thus associated with a reduced risk of OSCC (OR = 0.71; 95% CI 0.57–0.90, p = 0.004) (Table 1), with a p-value below the Bonferroni-corrected threshold of p = 0.0083. Further analysis of the *GSTT2B* polymorphism in the Mixed Ancestry population showed a stronger evidence for association under a dominant model of inheritance for the deletion (p = 0.002 for association), with carriers of the deletion allele having an OR of 0.57 (95% CI 0.40–0.81). The frequency of the *GSTT2B* deletion in Black controls (37%) was lower than that reported for a small Yoruban HapMap sample (47%), and the frequency in the Mixed Ancestry controls (50%) was lower than that reported for a Western European HapMap sample (63%) but identical to that for the Japanese/Chinese [22]. As with *GSTT1*, a case-only analysis did not detect any interaction between the *GSTT2B* deletion and smoking or alcohol use in either Black or Mixed Ancestry populations (Table 2), and interaction with smoking could not be tested in the Mixed Ancestry population because of the small number of non-smokers. However, in a case-control analysis of drinkers and smokers in the Mixed Ancestry population, the protective effect of the *GSTT2B* deletion was marginally stronger than in all cases (OR = 0.69 for both groups, p = 0.002 and 0.003 for smokers and drinkers respectively).

### *GSTP1* Ile105Val SNP (rs1695)

The genotype and allele frequencies obtained for the *GSTP1* Ile105Val SNP (rs1695) in the two populations is shown in Table 1. The 105Val allele was common in both populations, and genotype frequencies were in Hardy-Weinberg equilibrium in controls (p>0.05). This SNP was not associated with OSCC in either the Black or Mixed Ancestry populations. The frequency of the *GSTP1* Val105 allele in Black controls (53%) was marginally higher than that reported for African HapMap populations (36%–51%), but identical to that previously reported for South African Xhosa (53%) [27]; while in Mixed Ancestry controls it was slightly higher (44%) compared to Whites (41% in HapMap CEU). Case-only analysis did not detect any interaction between smoking or alcohol use and this SNP in either population group (Table 2). The non-smoker sample size in the Mixed Ancestry population (N = 13) was too low to perform an interaction test in this subgroup.

### Linkage disequilibrium analysis

Linkage disequilibrium (LD) between the *GSTT2B* and *GSTT1* deletion polymorphism was low in both populations. In the Black population, LD measures were r^2^ = 0.04 and D′ = 0.27. In the Mixed Ancestry population the *GSTT1* and *GSTT2B* deletions both have similar frequencies (0.495 and 0.501 respectively), but all four possible haplotypes exist, giving r^2^ = 0.07 and D′ = 0.33, with the *Del-Del* and *wildtype-wildtype* (*WT- WT*) haplotypes having lower frequencies than expected under linkage equilibrium. Haplotype analysis in the Black population showed no overall evidence of association (p = 0.500), and none of the individual haplotypes was associated with OSCC (Table 3). In the Mixed Ancestry population, strong evidence for association was obtained overall in the haplotype analysis (p = 0.0089), but this was due solely to the effect of *GSTT2B*, and conditional analysis showed that including *GSTT1* had no additional effect (p = 0.35). Examining the haplotype-specific results (Table 3) shows that the two haplotypes with a significant effect compared to the baseline *WT-WT* haplotype both carry the deletion at *GSTT2B* (*WT-DEL*, *DEL-DEL*), and have similar OR of 0.58, indicating that carrying an additional deletion at *GSTT1* did not reduce OSCC risk further. In Mixed Ancestry individuals, who were successfully genotyped for both the *GSTT1* and *GSTT2B* deletions, 13 of 414 controls (0.031) and 16 of 217 cases (0.074) were homozygous for both deletions (genotype *Del-Del*/*Del-Del*), which was a marginally significant difference (p = 0.027). In Black individuals this genotype was present in 22 of 462 controls (0.048) and 17 of 311 cases (0.055), which was not a significant difference (p = 0.786).

**Table 3:** Haplotype frequencies for the *GSTT1* and *GSTT2B* deletion polymorphisms in Black and Mixed Ancestry populations.

Population	Haplotype	Control Frequency		Linkage Disequilibrium			
	*GSTT1* – *GSTT2B*	Obs	Exp	r^2^	D′	OR	*p*-value
**Black**				0.04	0.27		
	*Wt – Wt*	0.2268	20.21			1 (reference)	-
	*DEL – Wt*	0.4004	d0.35			1.000 (0.74–1.36)	1.0000
	*Wt – DEL*	0.2223	e0.165			0.814 (0.56–1.18)	0.2796
	*DEL – DEL*	0.1505	30.28			0.912 (0.65–1.29)	0.6006
**Mixed Ancestry**				0.07	0.33		
	*Wt – Wt*	0.1711	0.25			1 (reference)	-
	*DEL – Wt*	0.3313	0.25			0.766 (0.52–1.12)	0.1705
	*Wt – DEL*	0.3337	0.25			0.584 (0.39–0.87)	0.0078
	*DEL – DEL*	0.1639	0.25			0.583 (0.39–0.88)	0.0083

**p-value is for comparing each haplotype to the reference** ***Wt-Wt*** **haplotype.**

## Discussion

In this study we have investigated the involvement of the *GSTT1* deletion, the *GSTP1* functional polymorphism Ile105Val and the recently described *GSTT2B* deletion, which greatly reduces expression of *GSTT2*, in susceptibility to OSCC in two South African populations. The *GSTT1* deletion was genotyped using a multiplex PCR assay which discriminates between subjects carrying zero, one or two functional alleles of the *GSTT1* gene [21]. Such individuals show a trimodal pattern of enzyme activity [39], thus discrimination between the 3 genotypes is important in the assessment of possible increased cancer risk in deletion heterozygotes. In our study, the *GSTT1* genotype showed no evidence of association with OSCC in either the Black or the Mixed Ancestry populations, which is consistent with the meta-analysis in White European and Asian populations [35]. Our analysis of the *GSTP1* Val105 allele also showed no association with OSCC. We did, however, observe association of the *GSTT2B* deletion with a reduced risk of OSCC in the Mixed Ancestry population (OR = 0.71; 95% CI 0.57–0.90). We did not find this association in the Black population; however the odds ratio (0.86) did overlap with the 95% confidence interval for the Mixed Ancestry population, so it is possible that a smaller effect might be revealed in the Black population in a larger sample size.

Power calculations show that our study is well-powered to identify association with variants that confer a direct association, and moderate power to detect a gene-environmental interaction. The Mixed Ancestry sample has 82% power to detect a genetic variant with frequency 0.5 (similar to the genotyped variants) which confers an increased odds ratio of 1.4 under an additive model. For the larger sample size in the Black population, there is a similar level of power (83%) to detect a lower odds ratio of 1.35. For the gene-environment interaction in a case-only analysis, an odds ratio of 1.75 would be detectable with power of 0.83 for the Mixed Ancestry population, and 94% for the Black population (assuming 30% exposure prevalence).

Oesophageal squamous cell carcinoma is a high-incidence cancer in South Africa, with smoking and alcohol drinking being important risk factors. We examined the interaction between smoking and alcohol use and the *GSTT1*, *GSTP1* and *GSTT2B* polymorphisms on risk of OSCC by testing for a difference in allele frequency between smokers and non-smokers, and between drinkers and non-drinkers (case-only analysis). We found no evidence for a different effect of the GST polymorphisms in smokers and non-smokers in either the Black or the Mixed Ancestry populations. Similarly, no interaction was detected with alcohol use.

The lack of an interaction betweeen the *GSTT2B* deletion and smoking or drinking in the Mixed Ancestry population may reflect the small numbers of cases in the non-smoking and non-drinking groups. For smoking, the number of non-smokers (N = 11) was too small to perform a meaningful statistical test. However, the protective effect of this deletion in smokers from the Mixed Ancestry population was marginally stronger than in all Mixed Ancestry cases.

The low level of linkage disequilibrium between *GSTT1* and *GSTT2B* which we observed in both the Black and Mixed Ancestry populations (r^2^<0.1) shows that the two deletions are independent and require separate assessment for disease risk in these populations. These results are broadly consistent with those of Zhao et al. [22], which reported strong LD between these deletions in Whites (r^2^ = 0.699), but weaker LD in the Japanese/Chinese (r^2^ = 0.173) and very low LD in Yorubans (r^2^ = 0.005). Also, haplotypes with both *GSTT1* and *GSTT2B* deletions were less frequent than expected in both South African populations (observed 5% and 16% vs 28% and 25% expected in Black and Mixed Ancestry populations respectively), which is consistent with the underrepresentation of the double-deletion haplotype in Whites [22]. The haplotype association test did not detect any association with OSCC in the Black population. The *GSTT1/GSTT2B DEL-DEL* haplotype was associated with reduced risk of OSCC in the Mixed Ancestry population, but the *GSTT1* deletion had no effect either alone or in combination with *GSTT2B*. It is interesting that a small proportion of both cases and controls are homozygously deleted for both *GSTT1* and *GSTT2B*, and are therefore likely to have little or no activity for the theta class of GST enzymes.

There are several possible explanations for the protective role of the *GSTT2B* deletion in OSCC. GSTs catalyze the conjugation of GSH to a wide variety of electrophilic compounds to produce water-soluble compounds which are excreted easily from the organism. GSH depletion to about 20–30% of total GSH levels may impair the conjugation defence against electrophilic compounds, which in turn could damage DNA and cause cancer initiation. Thus, the combined conjugation activities of GSTs may lead to GSH depletion and thereby increase cancer risk. Conversely, a decrease in GST activity as a result of deletion of some of the corresponding genes may produce an optimal GSH level to protect the organism against carcinogens. An alternative explanation may relate to the fact that GSTs, in addition to detoxification of carcinogens, can cause toxic effects in the cell. GSTs can convert several classes of compounds, via conjugation with GSH, into cytotoxic, genotoxic and mutagenic metabolites that can readily attack DNA and cause cancer initiation [16]. In this context, it is possible that the reduced *GSTT2* activity caused by the *GSTT2B* deletion may protect individuals from cancer. The fact that GSTs have overlapping substrate affinity is also relevant in this context. Thus deficiency of a single GST enzyme may not result in decreased detoxification of carcinogenic compounds because other GSTs with similar substrate specificities can compensate. The lack of association of *GSTT1* and *GSTP1* polymorphisms with OSCC observed in this study may reflect provision of their enzyme activity by other carcinogen-metabolizing enzymes. Simultaneous determination of the polymorphism of all the GSTs may therefore be an important prerequisite for reliable interpretation of the role of GST superfamily in susceptibility to OSCC. This could be addressed by development of a microarray that allowed genotyping of all known relevant polymorphisms in the GST and other gene families involved in the detoxification of carcinogens.

In conclusion, we have shown that the presence of the recently described *GSTT2B* deletion and the consequent reduction in *GSTT2* expression may have a protective effect on the initiation and development of oesophageal squamous cell carcinoma in the Mixed Ancestry South African population. It will be important to determine whether this effect can be replicated in other populations with a high incidence of OSCC, and to determine whether it contributes to susceptibility to other forms of cancer.

## Materials and Methods

### Study group

All patients diagnosed with OSCC were recruited from Groote Schuur Hospital in Cape Town, with the diagnosis confirmed by histopathological examination. Control samples were recruited from the healthy individuals from same population groups as the patients and from the same area, age-group, gender and ethnic group. Demographic information including ethnicity, language, gender, age, smoking and drinking habits was collected by interviews conducted by professional research nurses. This study was limited to Black and Mixed Ancestry South African individuals since they have a high prevalence of OSCC [7]. The Mixed Ancestry population was formed about 300 years ago from the union of different ethnic groups, receiving genetic contributions from the indigenous Khoi and San people and from Indonesian, European and sub-Saharan African populations [40]. A total of 1469 blood samples (907 controls and 562 cases) were collected. The control group included 479 Black and 428 Mixed Ancestry individuals, while the case group included 330 Black and 232 Mixed Ancestry individuals. Written informed consent was obtained from all control individuals and patients. Ethical approval for the study was obtained from the joint University of Cape Town/Groote Schuur Hospital Research Ethics Committee.

### Isolation and Purification of DNA

Blood samples were collected and stored at −20°C prior to DNA extraction. Genomic DNA was extracted according to the method of Gustafson et al. [41]. DNA concentration was determined by PicoGreen assay and the DNA samples were diluted to a concentration of 50 ng/µl for storage at −20°C.

### Determination of the GSTT1 and GSTT2B deletion genotypes

The *GSTT1* deletion polymorphism was genotyped by multiplex PCR assay as previously described [21]. A four primer set was used to identify both the deletion and non-deletion alleles in a single reaction (Fig. 2A). Briefly, 50 ng of genomic DNA was added to buffer containing 1.5 mM MgCl_2_, 2 mM dNTPs, 10 pmol of each oligonucleotide primer and 0.5 U of HotStart Taq polymerase, with GC-rich solution (Roche). Fragments were amplified in a final volume of 10 µl. The PCR reaction was carried out in an Applied Biosystems (Veriti Thermal Cycler) and thermal cycling conditions consisted of an initial hot start of 15 min at 95°C followed by 30 cycles of denaturation at 95°C for 30 sec, annealing at 65°C for 30 sec, and extension at 72°C for 1:30 min, with a final extension at 72°C for 7 min. Genotyping of the *GSTT2B* deletion polymorphism was performed by multiplex PCR assay using a three-primer set for simultaneous amplification of the non- deletion and deletion alleles (Fig. 2B), as previously described [22]. The PCR reaction was performed in a final volume of 10 µl with 0.5 U Taq polymerase (GoTaq, Promega), 1.5 mM MgCl_2_, 2 mM dNTPs, 10 pmol of each primer, and 50 ng of genomic DNA. The thermal cycling conditions consisted of an initial hot start of 95°C for 5 min, followed by 30 cycles of denaturation at 95°C for 30 sec, annealing at 55°C for 30 sec, and extension at 72°C for 60 sec. The final extension was at 72°C for 5 min. PCR products were separated by electrophoresis on 1% agarose gels in Tris-Borate-EDTA buffer. The gels were stained with ethidium bromide and digitally photographed using a UV transilluminator (Syngene Gel Documentation System, SN SYDR 4/2525). GeneRuler DNA Ladder Mix (Fermentas) was used as marker.

### Determination of the GSTP1 genotype

The *GSTP1* Ile105Val SNP (rs1695) was determined by TaqMan genotyping assay using fluorescent allele-specific TaqMan probes (reagents from Applied Biosystems). Amplification was performed in a total volume of 5 µl containing 2.5 of µl of TaqMan Master Mix (Applied Biosystems), 0,25 µl of SNP probe and 5 ng of DNA diluted in dH_2_O. The thermal cycling conditions consisted of an initial denaturation step at 95°C for 15 min, followed by 45 cycles of denaturation at 92°C for 15 min and annealing/extension at 60°C for 60 sec. Fluorescence was measured using a 7900HT Fast Real-Time PCR system and genotypes were assigned using SDS 2.2.2 software (Applied Biosystems). Successful genotyping was achieved for all 3 genetic variants in 99% of samples, with 12 samples classified as undetermined and not included in the study.

### Statistical analysis

The data were analyzed in a case-control design and the analysis was performed separating the Black and Mixed Ancestry population groups to avoid confounding effects due to ethnicity. Genotype counts among controls were tested for deviation from Hardy-Weinberg Equilibrium (HWE) using the chi-square test with one degree of freedom [42].

The genetic association study was performed assuming a multiplicative genetic model for any risk allele. We used a chi-square test on the allele-based 2×2 table, which tests for differences in allele frequency between cases and controls. Odds ratios (OR) and 95% confidence intervals (CI) were estimated for the tested allele. A conservative p-value threshold of 0.0083 ( = 0.05/(2*3)) was used for assessing significance, applying a Bonferroni correction for multiple testing of the two population groups and three polymorphisms tested. Significant results were followed up by investigation of dominant and recessive genetic models.

We performed case-only analysis of drinking and smoking by alleles using a 2×2 table of alleles against drinking or smoking status (yes/no). Where sample sizes were sufficiently large (≥40 cases in each group), we tested for differences in allele frequency using a chi-square test.

We determined the level of LD between the *GSTT1* and *GSTT2B* deletions, and tested for association of *GSTT1/GSTT2B* haplotypes with OSCC using UNPHASED [43].

Power calculations were carried out using Quanto, assuming a significance level of 0.05 (http://hydra.usc.edu/GxE).
